# Molecular Detection of *Candida auris* Using DiaSorin Molecular Simplexa^®^ Detection Kit: A Diagnostic Performance Evaluation

**DOI:** 10.3390/jof9080849

**Published:** 2023-08-15

**Authors:** Juan David Ramírez, Chin Yi Wang, Deandra Bolton, Bernadette Liggayu, Sarah Schaefer, Gopi Patel, Waleed Javaid, Carlos Cordon-Cardo, Adolfo Firpo-Betancourt, Emilia Mia Sordillo, Alberto Paniz-Mondolfi

**Affiliations:** 1 Department of Pathology, Molecular and Cell-Based Medicine, Icahn School of Medicine at Mount Sinai, New York, NY 10029, USA; chingyi.wang@mountsinai.org (C.Y.W.); deandra.bolton@mountsinai.org (D.B.); bernadette.liggayu@mountsinai.org (B.L.); carlos.cordon-cardo@mssm.edu (C.C.-C.); adolfo.firpo-betancourt@mountsinai.org (A.F.-B.); emilia.sordillo@mountsinai.org (E.M.S.); 2 Centro de Investigaciones en Microbiología y Biotecnología-CIMBIUR (UR), Facultad de Ciencias Naturales, Universidad del Rosario, Bogotá 200433, Colombia; 3 Division of Infectious Diseases, Department of Medicine, Icahn School of Medicine at Mount Sinai, New York, NY 10029, USA; sarah.schaefer@mountsinai.org (S.S.); gopi.patel@mountsinai.org (G.P.); waleed.javaid@mountsinai.org (W.J.)

**Keywords:** *Candida auris*, PCR, infection, sensitivity

## Abstract

*Candida auris* is a globally emerging fungal pathogen that is associated with healthcare-related infections. The accurate and rapid detection of *C. auris* is crucial for effective infection prevention, control, and patient management. This study aimed to validate the analytical and diagnostic performance of the DiaSorin Molecular *C. auris* Detection Kit. The analytical specificity, sensitivity, and reproducibility of the assay were evaluated. The limit of detection (LOD) was determined to be 266 CFU/µL using the ZeptoMetrix *Candida auris* Z485 strain and standard calibration curves. The assay demonstrated high analytical specificity and showed no amplification against a diverse panel of bacteria and fungi. Clinical validation was conducted using deidentified residual axillary/groin surveillance culture specimens from *C. auris* culture-positive and culture-negative patients. The DiaSorin Molecular Detection Kit exhibited 100% agreement in sensitivity, specificity, positive predictive value (PPV), and negative predictive value (NPV) when compared to cultures coupled with MALDI-TOF identification. Intra- and inter-reproducibility testing demonstrated consistent and reliable diagnostic performance. This validated assay offers rapid and accurate detection of *C. auris*, facilitating timely implementation of infection control measures and appropriate patient care. The DiaSorin Molecular *C. auris* Detection Kit has the potential to aid in controlling the outbreaks caused by this emerging fungal pathogen. Providing a reliable diagnostic tool can contribute to the effective management and containment of *C. auris* infections in healthcare settings and ultimately improve patient outcomes.

## 1. Introduction

*Candida auris* is an emerging fungal pathogen that has been reported in various regions of the world, including Asia, Europe, South America, and North America. The first documented case of *C. auris* infection was reported in Japan in 2009, and subsequently, the fungus rapidly disseminated globally [1]. *C. auris* is frequently associated with healthcare-associated infections, particularly among individuals with underlying medical conditions, prolonged hospitalization, or exposure to invasive medical devices [2]. The fungus can colonize diverse anatomical sites in patients, including the skin, gastrointestinal tract, and other mucosal surfaces, which poses challenges in terms of containment within healthcare facilities [3,4]. *Candida auris* was first documented in the United States in 2016 and has since emerged as a severe healthcare-associated infection. As reported by the Centers for Disease Control and Prevention (CDC) in January 2023, there have been more than 1800 confirmed cases of *C. auris* in the United States, with most cases originating in healthcare settings (CDC, 2023). *C. auris* infections have been identified in multiple states, including New York, New Jersey, Illinois, California, and Texas (CDC, 2023).

One of the most concerning features of *C. auris* is its high resistance to multiple classes of antifungal drugs, including azoles, echinocandins, and polyenes, which are commonly used in the treatment of fungal infections [5,6,7]. Some *C. auris* isolates have demonstrated resistance to all three major classes of antifungal drugs [5]. This resistance poses significant challenges in the management of *C. auris* infections and contributes to poor patient outcomes, highlighting the urgent need for effective control and treatment strategies.

Moreover, there is significant concern due to the occurrence of outbreaks of multidrug-resistant *Candida auris* infections in specific regions, including Florida (USA), India, and Germany, during 2020–2021. These outbreaks are potentially attributed to lapses in infection control and surveillance practices, influenced in part by the ongoing COVID-19 pandemic [8,9,10]. Recognizing the potential for outbreaks and the complexities associated with controlling *C. auris* infections, the Centers for Disease Control and Prevention (CDC) have categorized *C. auris* as an urgent threat to public health (CDC 2023). Ongoing surveillance and research are essential to gaining a comprehensive understanding of the epidemiology and transmission dynamics of this emerging fungal pathogen. Additionally, these efforts aimed to develop effective prevention and control strategies.

Diagnosing *Candida auris* infection presents challenges due to its non-specific clinical manifestations and resemblance in culture to other *Candida* species [11,12]. Furthermore, conventional diagnostic approaches, such as culture-based methods, are time-consuming and exhibit low sensitivity, particularly in patients with prior antifungal therapy. Matrix-assisted laser desorption/ionization time-of-flight mass spectrometry (MALDI-TOF MS) has emerged as a valuable tool for the rapid identification of *C. auris* [13,14], with the caveat that not all reference databases may allow for the accurate detection of *C. auris* species and strains from all its phylogenetic clades. Alternatively, molecular-based methods have demonstrated enhanced sensitivity and specificity in *C. auris* detection using polymerase chain reaction (PCR) and loop-mediated isothermal amplification (LAMP) assays, enabling direct detection of *C. auris* in a variety of clinical specimens such as blood, urine, respiratory secretions, or wound swabs [15,16].

Alternative approaches utilizing swabs from various anatomical regions have been proposed and have shown promising results [17,18,19]. Recently, DiaSorin Molecular introduced a set of primers designed to detect *C. auris* DNA using the Simplexa^®^ platform. However, to date, there have been no reports of the clinical validation of this kit or device. Therefore, in this study, we aimed to evaluate the performance of the DiaSorin Molecular Simplexa^®^ platform for *C. auris* detection. For this, we used a panel of deidentified *C. auris* axilla/groin surveillance specimens collected by ESwab™ in liquid Amies medium (BD Diagnostics) and compared the results of our molecular assay to those obtained using conventional culture-based identification methods.

Our study provides valuable insights into the reliability and accuracy of the DiaSorin Molecular Simplexa^®^ platform for detecting *C. auris*. Moreover, we underscore the potential utility of this platform in both clinical and public health settings. In addition, this study contributes to the growing body of knowledge regarding the implementation of molecular diagnostic tools for *C. auris* and supports the development of more effective strategies for timely detection and management of this emerging fungal pathogen.

## 2. Methods and Results

We employed the DiaSorin Molecular *C. auris* Detection Kit (Ref. MOL9059) to assess its analytical and diagnostic specificity, sensitivity, and reproducibility. The kit includes DiaSorin Molecular *C. auris* Primer Pair, which is an analyte-specific reagent designed to amplify a highly conserved region of the internal transcribed spacer 2 (ITS2) region of the rRNA gene of *Candida auris*. The primer pair consists of a forward primer, a reverse primer, and a FAM labeled probe, all in Tris-EDTA buffer. For proprietary reasons, the manufacturer does not disclose the specific sequence of the PCR target.

The ZeptoMetrix *Candida auris* Z485 strain (catalogue #0804386) (Zeptometrix^®^) was used to determine the analytical sensitivity. Each frozen aliquot contained 1 mL of a pure, titered culture of *Candida auris*, with organism identification confirmed by ITS2 sequencing as provided by the vendor. Standard calibration curves were generated using serial dilutions ranging from 2.6 × 10^6^ CFU/µL to 2.6 × 10^−3^ CFU/µL to establish the limit of detection (LOD). The dilutions were prepared using deidentified residual axilla/groin *C. auris* surveillance culture specimens that were culture-negative and were then spiked with the ZeptoMetrix *Candida auris* Z485 strain standard. All serially diluted spiked specimens were tested using the conditions specified in the DiaSorin Molecular Kit insert. The reproducibility and performance characteristics of the test were assessed, including the LOD, which provided crucial information on the sensitivity and reliability of the DiaSorin Molecular *C. auris* Detection Kit.

**Figure 1:**
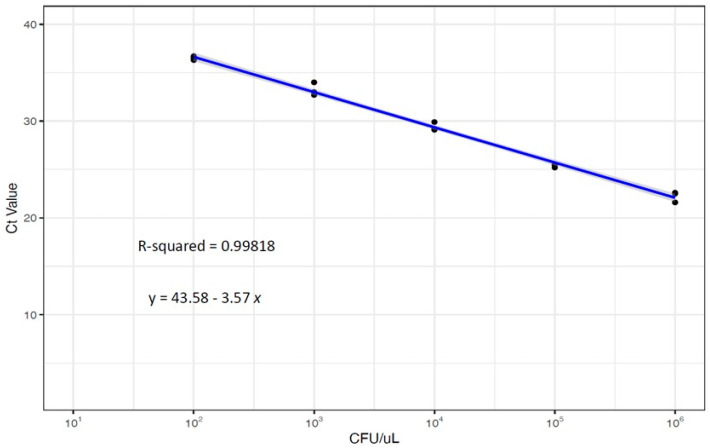
Dynamic range of Ct values for *C. auris* detection to determine the limit of detection (LOD).

The resulting Ct values of the calibration curve were plotted using linear regression and PROBIT regression to determine the limit of detection (LOD). The results indicated that the LOD, as determined by PROBIT regression, was 266 CFU/µL (Figure 1, Table 1). Furthermore, no significant differences were observed across the triplicates, indicating a high level of test reproducibility and calibration curve reliability (R^2^ > 0.980). Once the LOD was estimated, we conducted tests on three dilutions below the LOD that resulted in no amplification (Figure 1; Table 1). To assess the analytical specificity of the assay, we included a diverse panel of bacteria and fungi (Table 2), and no amplification was observed for any of the panels (Table 2).

The Clinical Microbiology Laboratory at Mount Sinai Health System routinely conducts microbiological diagnosis of *C. auris* using routine culture coupled with MALDI-TOF identification (Library 4.1.100 (PYTH) 188), as described by [20]. This combined approach serves as the gold standard for validation. To validate our molecular assay, we used deidentified residual axilla/groin specimens collected for surveillance culture for *C. auris* colonization using the ESwab™ in liquid Amies medium device. We evaluated deidentified ESwab™ specimens from 30 patients who were culture-positive for *C. auris* and 30 patients who tested culture-negative for *C. auris.* In all 60 cases, BD CultureSwab™ specimens had also been collected for culture. Routine culture and PCR (DiaSorin Molecular) results for all 60 samples and a comparison of both methodologies are presented in Table 3. In terms of diagnostic performance, we assessed sensitivity, specificity, positive predictive value (PPV), and negative predictive value (NPV) using culture results as the gold standard. The results showed 100% agreement and complete concordance for all the aforementioned values. In addition, subcultures from ESwab™-collected samples that were positive by the molecular assay led to the successful isolation of *C. auris* in 100% of the cases. This aspect is important as it highlights the potential dual role of the ESwab™: first as a suitable collection device for molecular identification, and secondly, as a reliable source for subsequent culture-based retrieval.

To evaluate the diagnostic reproducibility of the DiaSorin Molecular Detection Kit, we conducted intra-reproducibility testing using nine positive (Positive by culture and further confirmed with MALDI-TOF) and nine negative (Negative by culture) samples, each run in triplicate during the same run. Inter-reproducibility testing was performed using three positive and three negative samples, each run on three different days. The results of these reproducibility tests are presented in Supplementary Tables S1 and S2. No statistically significant differences were observed in the resulting Ct values between the inter- and intra-reproducibility tests, indicating a high level of diagnostic reproducibility of the assay.

## 3. Discussion

Here, we present an evaluation of the analytical and diagnostic specificity, sensitivity, and reproducibility of the DiaSorin Molecular *C. auris* Detection Kit. The analytical sensitivity, or limit of detection (LOD), was determined using standard calibration curves with cell-forming units of yeast (CFU). The results indicated that the LOD based on PROBIT regression ranged from 266 CFU/µL. Moreover, the assay demonstrated high reproducibility, with no statistically significant differences observed across triplicates. A diverse panel of bacteria and fungi was included to assess the analytical specificity of the assay, and the results revealed no amplification for any of the samples analyzed, indicating the ability of the assay to specifically detect *C. auris*.

Lastly, we evaluated diagnostic performance for the detection of *C.auris* in deidentified axilla/groin surveillance specimens. The main goal of this study was to assess the diagnostic performance of the DiaSorin Molecular *C. auris* Detection Kit using skin samples for pathogen surveillance. However, it is essential to consider the potential need for evaluating this kit’s performance in blood and serum samples in the future. As a result, we acknowledge this as a limitation of our study and emphasize it as a potential avenue for future research.

The culture, coupled with MALDI-TOF identification, served as the gold standard. The results showed 100% sensitivity, specificity, positive predictive value (PPV), and negative predictive value (NPV), demonstrating the reliability of the DiaSorin Molecular *C. auris* Detection Kit.

**Figure 2:**
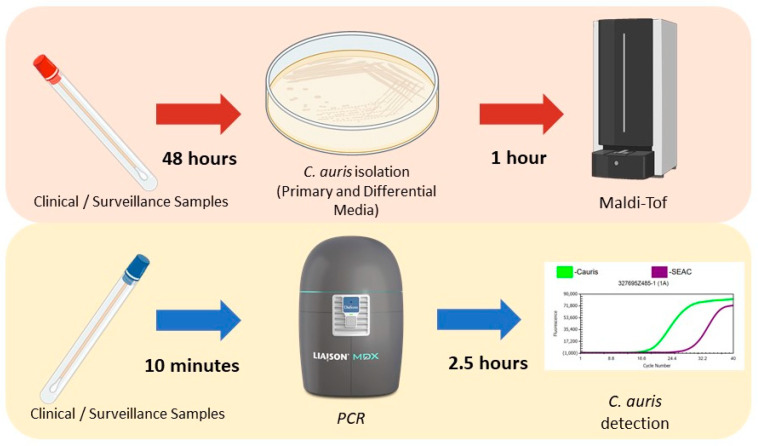
*Candida auris* diagnostic algorithm depicting testing method workflow differences and their impact on in-laboratory turn-around times. In red is highlighted the routine diagnostic workflow, and in yellow is the proposed workflow using the DiaSorin detection kit.

When reviewing the available literature on direct PCR methods utilizing various sample types such as swabs, sponges, sputum, urine, and others, we found that most authors reported a limit of detection (LOD) ranging between 1 and 54 CFU/reaction, using genetic markers such as ITS2, ITS1/2, GPI, and Pyruvate synthase [21]. In our study, we determined an LOD of 266 CFU/µL, which is equivalent to approximately 26 CFU/reaction, indicating sufficient analytical sensitivity (Figure 1 and Table 1). Additionally, the clinical advantage offered by the DiaSorin Molecular assay lies in its ability to bypass the need for culturing and DNA extraction, a crucial step in other commercial and in-house PCR and routine clinical assays (Figure 2) [17,22]. This characteristic makes the implementation of the DiaSorin Molecular Kit in clinical settings more convenient, especially for *C. auris* outbreak surveillance.

Various in-house and commercial platforms have been developed to detect *C. auris* nucleic acids, demonstrating satisfactory sensitivity (89–100%) and specificity (85–100%) [23,24]. Compared to the reference method of culture coupled with MALDI-TOF identification, this assay exhibits high diagnostic accuracy, indicating its broad utility for rapid surveillance and diagnosis. The platform delivered results within two hours of swab collection, further enhancing its clinical utility. Additionally, no cross-reactivity with a wide range of commensals and pathogens was observed (Table 2), which is often a limitation for other PCR platforms [23].

In conclusion, the DiaSorin Molecular *C. auris* Detection Kit exhibited outstanding analytical and diagnostic performances, demonstrating exceptional sensitivity, specificity, and reproducibility. The remarkable diagnostic accuracy of the assay establishes it as a valuable tool for the detection of *C. auris*. The potential applications of this kit are extensive, making it a valuable tool for rapid surveillance of *C. auris* in both skin and healthcare settings. Moreover, its affordability and easy accessibility through the DiaSorin vendor further enhance its appeal. It is important to note that while this test is not FDA-approved, our laboratory has diligently evaluated its diagnostic performance and subsequently submitted the assay as an LDT (laboratory developed test) to the New York State Department of Health, USA, for clinical testing.

We would like to draw attention to some limitations of the assay. Firstly, we were unable to determine if the test could detect all the *C. auris* clades due to the manufacturer not disclosing the specific sequence of the PCR target. Additionally, we were unable to test all known *Candida* species, such as *C. haemulonii*. It is crucial for future studies to address this limitation by including more species for testing specificity.

Given that *C. auris* is a significant healthcare-associated infection necessitating prompt and precise diagnosis for effective patient management, the capabilities of this assay are of immense clinical value. Future efforts should now be directed towards designing multiplex assays that incorporate targets for antifungal resistance mutations. These assays would enable rapid screening of drug-resistant *C. auris*.

## Figures and Tables

**Table 1:** Ct results for *C. auris* detection and internal control (SEAC) using Eswab as a matrix.

*C. auris* Dilution	*C. auris* (FAM)	SEAC (Q670)	*C. auris* (FAM)	SEAC (Q670)	*C. auris* (FAM)	SEAC (Q670)
2.66 × 10^6^ CFU/µL	21.6	31.4	22.6	32.6	22.5	31.6
2.66 × 10^5^ CFU/µL	25.2	30.3	25.5	31.5	25.5	30.5
2.66 × 10^4^ CFU/µL	29.2	30.6	29.9	31.4	29.1	30.7
2.66 × 10^3^ CFU/µL	34	31.4	33	32.1	32.7	31
2.66 × 10^2^ CFU/µL	36.7	31.4	36.3	32.2	36.5	31.2
2.66 × 10^1^ CFU/µL	0	31.7	0	32.1	0	31.3
2.66 × 10^0^ CFU/µL	0	31.5	0	32.4	0	31.5
2.66 × 10^−1^ CFU/µL	0	31.5	0	32.3	0	31.5
2.66 × 10^−2^ CFU µL	0	31.3	0	31.9	0	31.2
2.66 × 10^−3^ CFU/µL	0	31.5	0	32.1	0	31.2

**Table 2:** Ct results for *C. auris* detection over several microorganisms tested.

Sample ID	Organism	*C. auris* (FAM)	SEAC (Q670)	Result
CAIDNA001	*Staphylococcus aureus*	NaN	31.5	Not Detected
CAIDNA002	*Moraxella catarrhalis*	NaN	31.7	Not Detected
CAIDNA003	*Nocardia farcinica*	NaN	31.6	Not Detected
CAIDNA004	*Streptococcus immitis*	NaN	31.7	Not Detected
CAIDNA005	*Candida lusitaniae*	NaN	31.3	Not Detected
CAIDNA006	*Candida kefyr*	NaN	31.8	Not Detected
CAIDNA007	*Candida tropicalis*	NaN	31.7	Not Detected
CAIDNA008	*Candida guillermondi*	NaN	32.4	Not Detected
CAIDNA009	*Candida krusei*	NaN	32.5	Not Detected
CAIDNA010	*Candida parapsilosis*	NaN	31.6	Not Detected
CAIDNA011	*Candida glabrata*	NaN	31.5	Not Detected
CAIDNA012	*Phaeoanellomyces werneckii*	NaN	31.5	Not Detected
CAIDNA014	*Tricophyton rubrum*	NaN	32.1	Not Detected
CAIDNA015	*T. tonsurans*	NaN	32	Not Detected
CAIDNA016	*Fusarium*	NaN	32.1	Not Detected
CAIDNA019	*Penicillium*	NaN	31.6	Not Detected
CAIDNA020	*Aspergillus niger*	NaN	31.5	Not Detected
CAIDNA021	*Propionibacterium acnes*	NaN	31.6	Not Detected
CAIDNA022	*Bacillus cereus*	NaN	32	Not Detected
CAIDNA023	*M. abscessus*	NaN	31.6	Not Detected
CAIDNA024	*M. fortuitum*	NaN	31.9	Not Detected
CAIDNA025	*MAC*	NaN	32.3	Not Detected
CAIDNA026	*Mycobacterium chelonae*	NaN	31.4	Not Detected
CAIDNA027	*Enterococcus faecium*	NaN	31.6	Not Detected

**Table 3:** Resulting Ct for the 60 samples used for the validation.

Number	Sample ID	*C. auris* (FAM) Ct	SEAC (Q670) Ct	PCR Result	Culture Result
1	CA001	31.5	31	Detected	Detected
2	CA002	32.1	29.5	Detected	Detected
3	CA003	26.8	28.2	Detected	Detected
4	CA004	36.6	28.3	Detected	Detected
5	CA005	29.6	27.7	Detected	Detected
6	CA006	28.3	26.1	Detected	Detected
7	CA007	28.1	27.4	Detected	Detected
8	CA008	25.9	29.3	Detected	Detected
9	CA009	39	26.8	Detected	Detected
10	CA010	26.1	27.4	Detected	Detected
11	CA011	24.2	26.9	Detected	Detected
12	CA012	33.9	27.9	Detected	Detected
13	CA014	30.6	28.6	Detected	Detected
14	CA016	22.4	28.1	Detected	Detected
15	CA022	25	27.5	Detected	Detected
16	CA023	21.2	28.2	Detected	Detected
17	CA024	37.4	28.9	Detected	Detected
18	CA025	23.2	27.5	Detected	Detected
19	CA026	37.6	30.1	Detected	Detected
20	CA047	22.4	27.6	Detected	Detected
21	CA059	23.1	29.6	Detected	Detected
22	CA060	19.3	31.3	Detected	Detected
23	CA061	26.5	30	Detected	Detected
24	CA062	20.2	31.6	Detected	Detected
25	CA063	32.5	29.7	Detected	Detected
26	CA064	28.3	29.5	Detected	Detected
27	CA065	23	29.7	Detected	Detected
28	CA066	26	29.7	Detected	Detected
29	CA070	20.3	31.3	Detected	Detected
30	CA077	28.1	29.6	Detected	Detected
31	CA082	30.9	29.5	Detected	Detected
32	CA021	NaN	28.3	Not Detected	Not Detected
33	CA027	NaN	28	Not Detected	Not Detected
34	CA028	NaN	27.8	Not Detected	Not Detected
35	CA029	NaN	28	Not Detected	Not Detected
36	CA030	NaN	27.9	Not Detected	Not Detected
37	CA031	NaN	28	Not Detected	Not Detected
38	CA032	NaN	29.2	Not Detected	Not Detected
39	CA033	NaN	27.3	Not Detected	Not Detected
40	CA034	NaN	27.8	Not Detected	Not Detected
41	CA035	NaN	27.5	Not Detected	Not Detected
42	CA036	NaN	27.6	Not Detected	Not Detected
43	CA037	NaN	27.8	Not Detected	Not Detected
44	CA038	NaN	27.9	Not Detected	Not Detected
45	CA039	NaN	27.8	Not Detected	Not Detected
46	CA040	NaN	28	Not Detected	Not Detected
47	CA041	NaN	29.6	Not Detected	Not Detected
48	CA042	NaN	27.6	Not Detected	Not Detected
49	CA043	NaN	27.5	Not Detected	Not Detected
50	CA044	NaN	28	Not Detected	Not Detected
51	CA045	NaN	27.9	Not Detected	Not Detected
52	CA046	NaN	29.6	Not Detected	Not Detected
53	CA049	NaN	27.8	Not Detected	Not Detected
54	CA050	NaN	27.7	Not Detected	Not Detected
55	CA051	NaN	29.5	Not Detected	Not Detected
56	CA055	NaN	29.8	Not Detected	Not Detected
57	CA068	NaN	29.7	Not Detected	Not Detected
58	CA067	NaN	29.8	Not Detected	Not Detected
59	CA069	NaN	29.4	Not Detected	Not Detected
60	CA071	NaN	29.3	Not Detected	Not Detected
61	CA072	NaN	29.8	Not Detected	Not Detected

## Data Availability

All data is within the manuscript and the Supporting Files.

## Supplementary Materials

The following supporting information can be downloaded at: https://www.mdpi.com/article/10.3390/jof9080849/s1. Table S1: Intra-run Ct results for tested specimens; Table S2: Inter-run Ct results for tested specimens.

## References

[1] Spivak E.S., Hanson K.E. (2018). *Candida auris*: An Emerging Fungal Pathogen. J. Clin. Microbiol..

[2] Tsay S., Welsh R.M., Adams E.H., Chow N.A., Gade L., Berkow E.L., Poirot E., Lutterloh E., Quinn M., Chaturvedi S. (2017). Notes from the Field: Ongoing Transmission of *Candida auris* in Health Care Facilities—United States, June 2016–May 2017. MMWR Morb. Mortal. Wkly. Rep..

[3] Satoh K., Makimura K., Hasumi Y., Nishiyama Y., Uchida K., Yamaguchi H. (2009). *Candida auris* sp. nov., a novel ascomycetous yeast isolated from the external ear canal of an inpatient in a Japanese hospital. Microbiol. Immunol..

[4] Zaoutis T.E., Argon J., Chu J., Berlin J.A., Walsh T.J., Feudtner C. (2005). The Epidemiology and Attributable Outcomes of Candidemia in Adults and Children Hospitalized in the United States: A Propensity Analysis. Clin. Infect. Dis..

[5] Jacobs S.E., Jacobs J.L., Dennis E.K., Taimur S., Rana M., Patel D., Gitman M., Patel G., Schaefer S., Iyer K. (2022). *Candida auris* Pan-Drug-Resistant to Four Classes of Antifungal Agents. Antimicrob. Agents Chemother..

[6] Lockhart S.R., Etienne K.A., Vallabhaneni S., Farooqi J., Chowdhary A., Govender N.P., Colombo A.L., Calvo B., Cuomo C.A., Desjardins C.A. (2017). Simultaneous Emergence of Multidrug-Resistant *Candida auris* on 3 Continents Confirmed by Whole-Genome Sequencing and Epidemiological Analyses. Clin. Infect. Dis..

[7] Vallabhaneni S., Kallen A., Tsay S., Chow N., Welsh R., Kerins J., Kemble S.K., Pacilli M., Black S.R., Landon E. (2017). Investigation of the First Seven Reported Cases of *Candida auris*, a Globally Emerging Invasive, Multidrug-Resistant Fungus-United States, May 2013–August 2016. Am. J. Transplant..

[8] Janniger E.J., Kapila R. (2021). Public health issues with *Candida auris* in COVID-19 patients. World Med. Health Policy.

[9] Hinrichs C., Wiese-Posselt M., Graf B., Geffers C., Weikert B., Enghard P., Aldejohann A., Schrauder A., Knaust A., Eckardt K. (2022). Successful control of *Candida auris* transmission in a German COVID-19 intensive care unit. Mycoses.

[10] Rajni E., Singh A., Tarai B., Jain K., Shankar R., Pawar K., Mamoria V., Chowdhary A. (2021). A High Frequency of *Candida auris* Blood Stream Infections in Coronavirus Disease 2019 Patients Admitted to Intensive Care Units, Northwestern India: A Case Control Study. Open Forum Infect. Dis..

[11] Kathuria S., Singh P.K., Sharma C., Prakash A., Masih A., Kumar A., Meis J.F., Chowdhary A. (2015). Multidrug-Resistant *Candida auris* Misidentified as Candida haemulonii: Characterization by Matrix-Assisted Laser Desorption Ionization—Time of Flight Mass Spectrometry and DNA Sequencing and Its Antifungal Susceptibility Profile Variability by Vitek 2, CLSI Broth Microdilution, and Etest Method. J. Clin. Microbiol..

[12] Diezmann S., Cox C.J., SchÖnian G., Vilgalys R.J., Mitchell T.G. (2004). Phylogeny and evolution of medical species of Candida and related taxa: A multigenic analysis. J. Clin. Microbiol..

[13] Grenfell R.C., Junior A.R.d.S., Del Negro G.M.B., Munhoz R.B., Gimenes V.M.F., Assis D.M., Rockstroh A.C., Motta A.L., Rossi F., Juliano L. (2016). Identification of Candida haemulonii Complex Species: Use of ClinProToolsTM to Overcome Limitations of the Bruker BiotyperTM, VITEK MSTM IVD, and VITEK MSTM RUO Databases. Front. Microbiol..

[14] Mizusawa M., Miller H., Green R., Lee R., Durante M., Perkins R., Hewitt C., Simner P.J., Carroll K.C., Hayden R.T. (2017). Can Multidrug-Resistant *Candida auris* Be Reliably Identified in Clinical Microbiology Laboratories?. J. Clin. Microbiol..

[15] Yamamoto M., Alshahni M.M., Tamura T., Satoh K., Iguchi S., Kikuchi K., Mimaki M., Makimura K. (2018). Rapid Detection of *Candida auris* Based on Loop-Mediated Isothermal Amplification (LAMP). J. Clin. Microbiol..

[16] Kordalewska M., Zhao Y., Lockhart S.R., Chowdhary A., Berrio I., Perlin D.S. (2017). Rapid and Accurate Molecular Identification of the Emerging Multidrug-Resistant Pathogen *Candida auris*. J. Clin. Microbiol..

[17] Walchak R.C., Buckwalter S.P., Zinsmaster N.M., Henn K.M., Johnson K.M., Koelsch J.M., Herring S.A., Steinmetz L.K., Reed K.A., Barth J.E. (2020). *Candida auris* Direct Detection from Surveillance Swabs, Blood, and Urine Using a Laboratory-Developed PCR Method. J. Fungi.

[18] Sexton D.J., Kordalewska M., Bentz M.L., Welsh R.M., Perlin D.S., Litvintseva A.P. (2018). Direct Detection of Emergent Fungal Pathogen *Candida auris* in Clinical Skin Swabs by SYBR Green-Based Quantitative PCR Assay. J. Clin. Microbiol..

[19] Bayona J.V.M., García C.S., Palop N.T., Cardona C.G. (2021). Validation and implementation of a commercial real-time PCR assay for direct detection of *Candida auris* from surveillance samples. Mycoses.

[20] Černáková L., Roudbary M., Brás S., Tafaj S., Rodrigues C.F. (2021). *Candida auris*: A Quick Review on Identification, Current Treatments, and Challenges. Int. J. Mol. Sci..

[21] Dennis E.K., Chaturvedi S., Chaturvedi V. (2021). So Many Diagnostic Tests, So Little Time: Review and Preview of *Candida auris* Testing in Clinical and Public Health Laboratories. Front. Microbiol..

[22] Leach L., Zhu Y., Chaturvedi S. (2018). Development and Validation of a Real-Time PCR Assay for Rapid Detection of *Candida auris* from Surveillance Samples. J. Clin. Microbiol..

[23] Lima A., Widen R., Vestal G., Uy D., Silbert S. (2019). A TaqMan Probe-Based Real-Time PCR Assay for the Rapid Identification of the Emerging Multidrug-Resistant Pathogen *Candida auris* on the BD Max System. J. Clin. Microbiol..

[24] Malczynski M., Dowllow N., Rezaeian S., Rios J., Dirnberger L., Zembower J.A., Zhu A., Qi C. (2020). Optimizing a real-time PCR assay for rapid detection of *Candida auris* in nasal and axillary/groin samples. J. Med. Microbiol..

