# Supplementary material for: Molecular Detection of Candida auris Using DiaSorin Molecular Simplexa® Detection Kit: A Diagnostic Performance Evaluation
Source: J Fungi (Basel). 2023 Aug 15;9(8):849. doi: 10.3390/jof9080849 (PMC10455898; doi:10.3390/jof9080849)
Supplement: Supplementary file 1 [file jof-09-00849-s001.zip › jof-2531205-supplementary.pdf]

**Table S1.** Intra-run Ct results for tested specimens

| Sample ID | *C. auris* (FAM) | SEAC (Q670) | Result |
|---|---|---|---|
| CA065a | 15 | 33.2 | Detected |
| CA065b | 18.8 | 31.3 | Detected |
| CA065c | 13.7 | 33.9 | Detected |
| CA066a | 17.6 | 31.1 | Detected |
| CA066b | 18.5 | 30.9 | Detected |
| CA066c | 17.3 | 32.2 | Detected |
| CA082a | 16.8 | 32.4 | Detected |
| CA082b | 18.6 | 31.5 | Detected |
| CA082c | 16.8 | 32.4 | Detected |
| CA093a | NaN | 30.4 | Not Detected |
| CA093b | NaN | 31 | Not Detected |
| CA093c | NaN | 31.4 | Not Detected |
| CA094a | NaN | 31 | Not Detected |
| CA094b | NaN | 31.1 | Not Detected |
| CA094c | NaN | 31.1 | Not Detected |
| CA096a | NaN | 31.2 | Not Detected |
| CA096b | NaN | 31 | Not Detected |
| CA096c | NaN | 30.9 | Not Detected |

**Table S2.** Inter-run Ct results for tested specimens

| Run 1 | C.auris (FAM) | SEAC (Q670) | Run 2 | C.auris (FAM) | SEAC (Q670) | Run 3 | C.auris (FAM) | SEAC (Q670) | Result |
|---|---|---|---|---|---|---|---|---|---|
| CA065a | 15 | 33.2 | CA065a | 13.7 | 35.1 | CA065a | 19.1 | 28.1 | Detected |
| CA066a | 17.6 | 31.1 | CA066a | 13 | 37.9 | CA066a | 19 | 28.3 | Detected |
| CA082a | 16.8 | 32.4 | CA082a | 18.6 | 31.4 | CA082a | 13.9 | 30.5 | Detected |
| CA093a | NaN | 30.4 | CA093a | NaN | 31.1 | CA093a | NaN | 28.2 | Not Detected |
| CA093c | NaN | 31.4 | CA093c | NaN | 31.1 | CA093c | NaN | 28.2 | Not Detected |
| CA094a | NaN | 31 | CA094a | NaN | 31 | CA094a | NaN | 28 | Not Detected |
